# Why call it developmental bias when it is just development?

**DOI:** 10.1186/s13062-020-00289-w

**Published:** 2021-01-09

**Authors:** Isaac Salazar-Ciudad

**Affiliations:** 1 Evo-devo Helsinki community, Centre of Excellence in Experimental and Computational Developmental Biology, Institute of Biotechnology, University of Helsinki, Helsinki, Finland; 2 Genomics, Bioinformatics and Evolution, Departament de Genètica i Microbiologia, Universitat Autònoma de Barcelona, Cerdanyola del Vallès, Spain; 3 Centre de Rercerca Matemàtica, Cerdanyola del Vallès, Spain

**Keywords:** Developmental bias, Morphological evolution, Development, Morphogenesis, Developmental mechanisms, Developmental constraints, Variational properties

## Abstract

The concept of developmental constraints has been central to understand the role of development in morphological evolution. Developmental constraints are classically defined as biases imposed by development on the distribution of morphological variation.

This opinion article argues that the concepts of developmental constraints and developmental biases do not accurately represent the role of development in evolution. The concept of developmental constraints was coined to oppose the view that natural selection is all-capable and to highlight the importance of development for understanding evolution. In the modern synthesis, natural selection was seen as the main factor determining the direction of morphological evolution. For that to be the case, morphological variation needs to be isotropic (i.e. equally possible in all directions). The proponents of the developmental constraint concept argued that development makes that some morphological variation is more likely than other (i.e. variation is not isotropic), and that, thus, development constraints evolution by precluding natural selection from being all-capable.

This article adds to the idea that development is not compatible with the isotropic expectation by arguing that, in fact, it could not be otherwise: there is no actual reason to expect that development could lead to isotropic morphological variation. It is then argued that, since the isotropic expectation is untenable, the role of development in evolution should not be understood as a departure from such an expectation. The role of development in evolution should be described in an exclusively positive way, as the process determining which directions of morphological variation are possible, instead of negatively, as a process precluding the existence of morphological variation we have no actual reason to expect.

This article discusses that this change of perspective is not a mere question of semantics: it leads to a different interpretation of the studies on developmental constraints and to a different research program in evolution and development. This program does not ask whether development constrains evolution. Instead it asks questions such as, for example, how different types of development lead to different types of morphological variation and, together with natural selection, determine the directions in which different lineages evolve.

## Background

One central tenet of evolutionary developmental biology, or evo-devo, is that development is important for understanding morphological evolution [1–11]. Each multicellular morphology is produced from some simple initial condition (e.g. a zygote) through a complex process of development. In this process changes in the position of cells and extracellular matrix (i.e. morphology) occur because cells, extracellular matrix (ECM) and gene products interact in complex dynamic networks [12, 13]. Understanding how these networks of interactions function, namely development, is important to understand morphology and how it varies due to genetic and environmental variations. Certainly, mutation and recombination determine variation at the genetic level, but it is development that determines the morphological variations that arise from genetic variations and, thus, the morphological variations that are possible in each generation and population [14, 15].

**Fig. 1:**
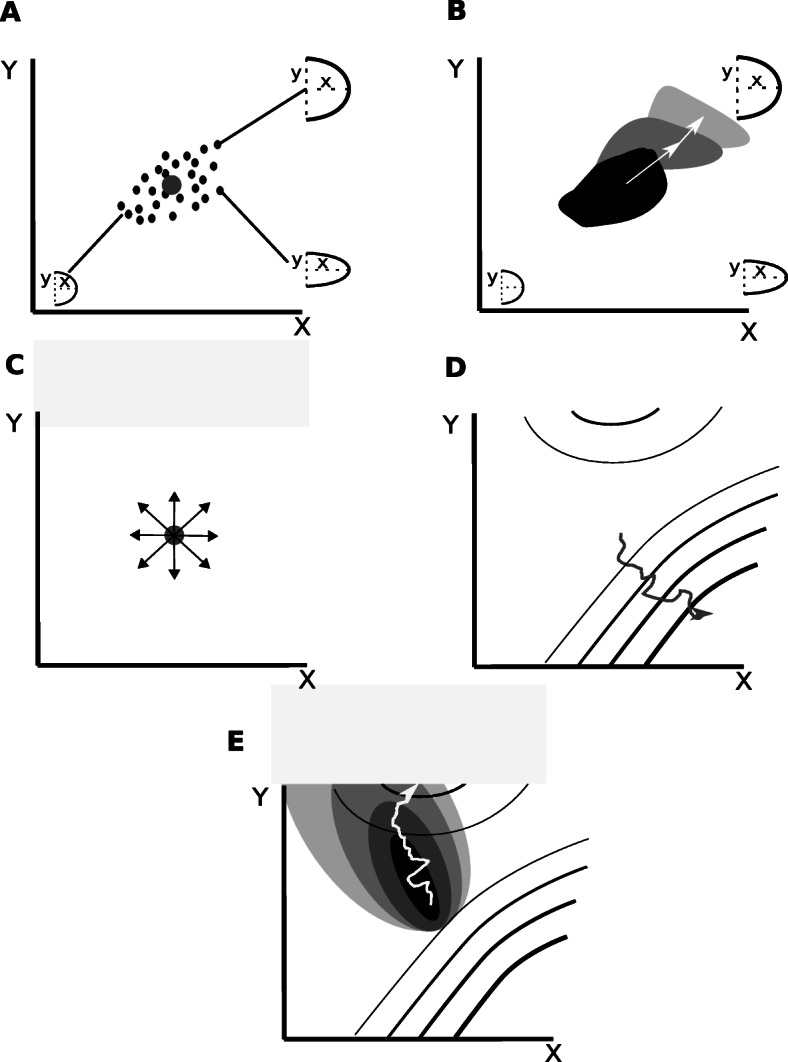
The direction of morphological evolution. **a** Example of a two-trait morphospace considering, for example, limb length (X-axis) and width (Y-axis). Each point in such morphospace represents an individual limb morphology in a population. The gray point represents the population mean for both traits. **b** Example of evolution in a morphological direction in the limb morphospace The gray areas represent the distribution of the individuals of a population at different successive generations. The arrows show the direction of evolution between generations (a vector from each generation mean to the next generation mean). **c** Arrows show a sample of the directions of possible morphological variation under the isotropic expectation for a two-trait morphospace. **d** Example of a fitness landscape on the same two-trait limb morphospace. The contour lines show points, i.e. limb morphologies, with the same fitnes (the higher the fitness the thicker the line). In this example morphological variation is isotropic so the population can go from any point in the morphospace to any other nearby point. As a result one can deduce how morphology will change based on the fitness landscape: the population would evolve towards the steepest peak. The gray line shows that this is the evolutionary trajectory the population would follow. **d** As in C but in this case morphological variation is not isotropic. The gray regions show the morphologies, i.e. points in the morphospace, that are possible by changes in development (the darker the point the more likely it is to arise from changes in development). In this case, as shown by the trajectory in white, the population would not evolve towards the steepest fitness peak because there is no morphological variation in that direction

Natural selection can only act on existing phenotypic variations [3, 16–18]. From an evo-devo perspective, thus, both development and natural selection are crucial in determining the direction of morphological evolution: development would “propose” a set of possible morphological variants in each generation and natural selection would choose which of them pass to the next generation. In this article “the direction of morphological evolution” is understood as the specific way in which morphology changes between generations in evolution. For example, if one represents a morphology by a set of quantitative traits, as in Fig. 1 (see Fig. 1a-c), then the direction of morphological evolution is a vector pointing from each trait’s mean in one generation to each trait’s mean in another generation in a population.

This opinion article argues that the concepts of developmental constraints and developmental biases do not accurately represent the role of development in evolution. This article discusses the assumptions on which development was first described as a constraint or bias in evolution (sections 1 and 2), and why these assumptions should no longer be regarded as tenable (sections 3 to 6). From that I argue that, although these concepts were coined to highlight the importance of development in morphological evolution [3, 10, 14], they do not fully capture the fact that it is development that determines possible morphological variation and the possible directions of evolution (section 8). Section 8 also proposes how to conceptualize the role of development in evolution without the shortcomings of the developmental constraints and bias concepts. Section 9 discusses the relationship between the concepts introduced in section 8 and the concept of evolvability. Section 10 provides concrete examples of the shortcomings of the developmental constraints and biases in experimental research. Section 11 describes how the alternative concepts I propose lead to a different research program.

## Developmental bias as a departure from an expected morphological distribution

The concept of developmental constraints is perhaps the most commonly used concept to describe the role of development in evolution [3, 4, 8, 10, 14, 15, 18–22]. This concept has generated substantial controversy over the years. This controversy touches on topics such as the nature of developmental constraints, their importance or even their existence. Such controversy ultimately relates to how we understand development, its relationship to morphological variation and the relationship between those two and evolution [5, 8, 18, 21, 23–27].

Gould’s work provides one of the most influential discussions on constraints in evolutionary biology [28–30]. Gould [19] understands constraints as:

*“the sources of changes, or restrictions upon change, that do not arise through the action of stated causes within a favored theory”*

For Gould, the alleged favored theory was the modern synthesis and what he calls its functionalist approach to explaining form. The “sources of changes or restrictions upon change” is, in the case of developmental constraints, development [19]. In brief, development is seen as a constraint because it is seen as precluding morphological evolution from being explainable from natural selection alone. According to Gould [19], of the many reasons for constraint in evolution, development must rank first.

Following Gould’s ideas and an improved understanding of developmental biology, later authors provided a more detailed discussion on how development can constrain evolution [3, 4, 8, 10, 14, 15, 18–22]. A consensus definition of developmental constraints is [5]: “A bias imposed on the distribution of phenotypic variation arising from the structure, character, composition or dynamics of the developmental system”. Notice that developmental bias was already included in the definition of developmental constraints. Some authors [27], however, make a distinction between developmental bias and developmental constraints. The difference between these two concepts is a matter of degree: developmental bias describes that, due to development, not all morphological variation is equally likely while developmental constraint describes that, due to development, some morphological variation is not possible (in a population, species, etc.). As we will see, the discussion in this article applies to both developmental bias and developmental constraints. Most of the literature on developmental bias and constraints focuses in morphology and, thus, the discussion in this article applies, primarily, to morphology.

In a scientific context, a bias is generally understood as a difference between the observed and expected values in some property of a distribution [31]. For example, an estimator of the mean in a population is called biased if it departs systematically from the actual population mean (e.g. it is lower than it). Therefore, the classical definition of developmental bias says that there is a developmental bias if an observed distribution of morphological phenotypes (e.g. in a population or species) is different from an expected distribution, and this difference is due to the “structure, character, composition or dynamics of the developmental system” [5]. In other words, development is seen as producing a departure from an expected distribution of morphological variation. But which is this expected distribution? And how can development be the reason for a bias on expected morphological variation if, as asserted in the introduction, it is development that determines which morphological variation is possible (i.e. the morphological distribution itself)?

## What is the expected distribution

One main intention of the proponents of the developmental constraints concept was to argue that development is important for understanding evolution [3, 5, 15]. This idea had to be defended because it was foreign to most evolutionary biologists. The prevailing evolutionary theory at the time, and to some extent nowadays, was the modern synthesis [32]. The modern synthesis did not consider development to be important [3, 17]. The view was that natural selection is the only fundamental process determining the direction of evolutionary change and that other processes play either no fundamental role (e.g. development) or play no directional role (e.g. genetic drift). However, natural selection can only act on existing phenotypic variation. Then, for natural selection to be the only important factor determining the direction of evolution, it is required that morphological variation is possible and equally likely in all directions [3, 9, 14, 19], at least by the small gradual changes favored in the modern synthesis. If that is not the case, a given population may not necessarily evolve into the direction most favored by natural selection. This is because there may not be individuals exhibiting morphological variation in such direction (see Fig. 1d-e for more detail). Instead, the population would evolve into another, less adaptive direction in which morphological variation is likely, or at least possible [3, 9, 14] (see Fig. 1e). The direction of morphological evolution, then, is not determined by natural selection alone: the processes that determine which phenotypic variations can arise by mutation, e.g. development, also play a role in determining the directions of morphological evolution.

In this article, I use the term *the isotropic expectation* for the expectation that morphology can vary in any direction and with equal probability. In other words, any trait can vary and all traits have the same probability to do so (see Fig. 1c).

To the best of my knowledge, the isotropic expectation has rarely been explicitly stated (however see [33, 34]). The isotropic expectation is simply a logical requirement for the argument that natural selection is the only important factor determining the direction of morphological evolution. What is sometimes claimed is that variation is random and gradual [35]. There are several meanings of the word random in evolutionary biology [30, 35]. If “random” is used to argue that natural selection is the only important factor determining the direction of evolution, then “random” is clearly equivalent to “isotropic” [3].

## Development is not the reason why morphological variation is not possible in all directions

The original proponents of the developmental constraints concept defended the idea, as here, that development is the process determining possible morphological variation [3, 14]. However, characterizing the role of development in evolution as a bias or a constraint seems to imply that development is the reason why not all morphological variation is possible or equally likely. When discussing developmental constraints, Alberch [14] states that:

*“Nevertheless, if it can be shown that the structure of the genetic and epigenetic system can constrain the amount of expressed phenotypic variation resulting from random genetic mutations and environmental perturbation, then, I will argue, the potential pathways of transformation become finite and an additional deterministic component is imposed on evolutionary processes by the structure of the developmental program”.*

In this statement Alberch argues that development, i.e. “the structure of the genetic and epigenetic system”, constrains the expression of phenotypic variation that would otherwise result from “genetic mutations and environmental perturbations” and that, because of that, “the potential pathways of transformation become finite”. This seems to imply that development is the reason why the potential pathways of transformation become finite, somehow as opposed to the possibility of being infinite. This statement also seems to imply that random genetic mutations have some sort of default morphological effects that are precluded by development.

The view that morphological variation is isotropic and that genes have inherent morphological effects was very prevalent at the time the developmental constraints concept was coined (and even later, see Wade’s discussion [36]). As discussed by Wade and others [36, 37], these views are rarely stated explicitly in the literature. Instead, they are implicitly assumed when arguing about morphological evolution, especially in the modern synthesis. Thus, the above quoted statement should not be interpreted as what Alberch and colleagues thought, but as a didactic way for them to explain the importance of development to those holding the isotropic expectation. Alberch and colleagues may have chosen to conceptualize the role of development in evolution by taking these views as the starting point or expectation and, then, describing development as the reason for the non-fulfillment of these expectations. In other words, the role of development in evolution was described as a constraint in respect to what would be expected from the isotropic expectation necessary for natural selection to be the only important factor in morphological evolution.

Although Alberch and colleagues [3, 14] defended the idea, as here, that it is development that determines possible morphological variation, the way they did it is somehow contradictory: Describing the role of development as precluding some morphological variation from arising is not really compatible with the fact that morphology arises because of development (i.e. the interactions between cells, ECM and genes during development). Multicellular morphology has to be constructed through cell, ECM and gene interactions in order to exist and, thus, genetic variation has an effect on morphology only because it affects these interactions, i.e. development. In other words, without gene, cell and ECM interactions, i.e. without development, there is no multicellular morphology, no morphological variation and, thus, no morphological evolution. Development is, thus, not a constraint or bias. Development is, instead, the reason why morphological variation is possible in some directions in the first place and, thus, the reason why evolution can eventually happen in those directions.

## On the question of why some morphologies do not exist

The concepts of developmental constraints and bias naturally lead to ask why are some morphologies observed more often than others and why are some not observed at all (e.g. in a population, a species, a group of species). For example, Alberch asks [14]:

*“How do we explain the empty spaces and the ordered pattern in morphology-space?”.*

Here Alberch is asking why some morphologies are not observed in nature: the “empty spaces”. However, the number of unobserved morphologies one can ask about is infinite, therefore, it only makes sense to ask for the non-existence of specific ones if there are, first, some reasons to expect these should exist in nature.

One possible reason to expect the existence of some morphologies is development. Some hypotheses about how development works in a species may lead to predict that some morphologies could exist (e.g. in a population, species, etc.). In this case, however, development cannot be seen as a constraint or bias on an expectation on possible morphology since development is, explicitly, the source of such expectation.

Another possible reason to expect the existence of some morphologies would be, as explained above, the isotropic expectation required for natural selection to be the only important factor determining the direction of morphological evolution. The next two sections explain that this expectation is untenable. If the isotropic expectation is untenable, then, it does not make sense to ask why some morphologies do not exist since, simply, there is no reason to expect them to exist to start with. Naturally, it does not make sense either to blame development for the non-existence of those morphologies and, then, claim that they are developmentally constrained or biased.

## The isotropic expectation and quantitative genetics

Quantitative and population genetics are usually regarded as the core of the modern synthesis [35, 38]. There is a large body of experimental research showing that most phenotypic traits readily respond to artificial selection [39, 40]. This research shows that many traits exhibit additive genetic variation. Additive genetic variation is phenotypic variation that can be approximated as arising from alleles whose phenotypic effects are independent from the environment and other alleles and loci. Since most traits respond to natural selection, it is argued that there is always abundant variation on which natural selection can act [23, 24, 41, 42] and, thus, that natural selection can always act. However, that natural selection can always act does not mean that evolution would necessarily happen in the direction most favored by natural selection or, equivalently, that natural selection is the only important factor determining the direction of morphological evolution. For example, natural selection may be favoring a specific proportion between two or more traits (e.g. limbs that are long and thin) but limbs with those proportions may not be possible, or likely, in limb development. In other words, development may not be able to produce limbs that are very long and, at the same time, very thin (even if they happen to be very adaptive). Evolution may then proceed to either long but not-so-thin limbs or to thin but not-so-long limbs.

There are only a handful of studies directly exploring which directions of morphological variation are possible from a given morphology. These studies measure many traits in a natural population and estimate in which directions there is variation [43, 44]. These studies found that there is variation in many but not all phenotypic directions.

## The isotropic expectation and current developmental biology

The isotropic expectation is not based on any understanding of development [13] or molecular biology [32]. To my knowledge, nobody has ever proposed how development could possibly work to produce a morphology and variation in all directions from it (Fig. 1). The original literature on developmental constraints already explained that what was known about development did not support the view that morphological variation should be possible in all directions [3, 5, 14]. Here, I add to these early explanations a set of arguments that, based on current developmental biology, indicate that it could not be otherwise: there is no way in which development could lead to morphological variation being possible in all directions.

The development of an organism can be understood as a sequence of morphological transformations going from a zygote to an adult morphology. For convenience we can divide development into a set of discrete transformations between morphologies (i.e. specific distributions of cells in space). For example, development is typically studied from some arbitrary “initial” morphology, (e.g. a primordial limb bud in the side of the embryo) to a later arbitrary “final” morphology (e.g. the adult limb).

**Fig. 2:**
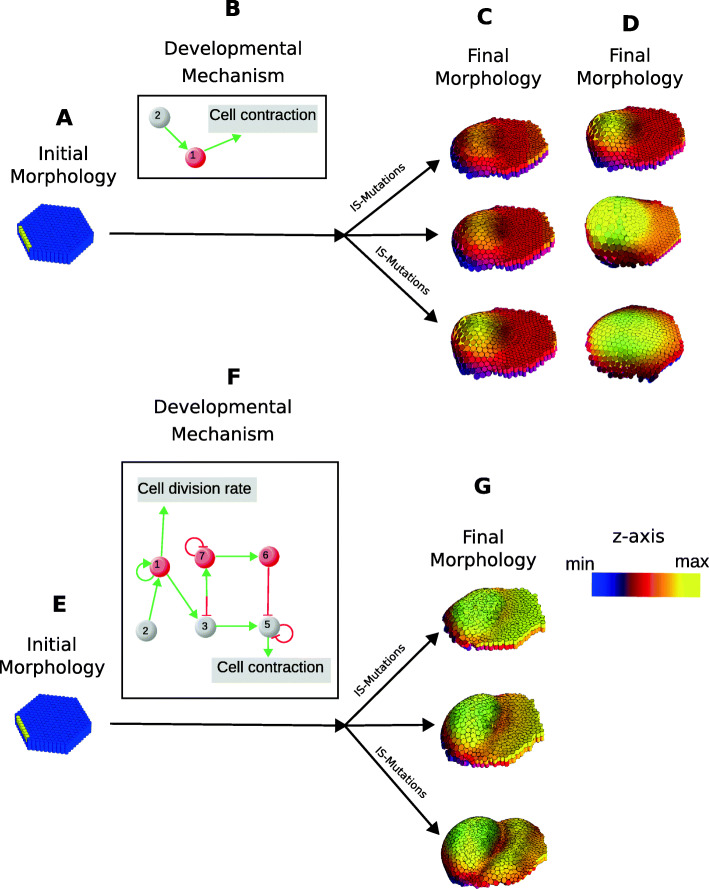
Developmental mechanisms, initial and final morphology. **a** Example of an initial morphology. Cylinders represent epithelial cells. Cells in yellow express a gene that is not expressed in the cells in blue. All blue cells express the same genes. **b** Example of a developmental mechanism. Balls represent gene products. Red balls are extracellular diffusive gene products (a signal). Gene 2 is the gene expressed by the cells in yellow in **a**. For simplicity the signal transduction pathway is not represented. Green arrows represent positive regulation. Red cells represent negative regulation. Squares represent cell behaviors or cell mechanical properties. **c** Three examples of final morphologies arising from the initial morphology in **a** through the developmental mechanism in **b** (according to a mathematical general model of development called EmbryoMaker [45]). The morphological variation is the one arising from variation in the amount of signal being secreted (the signal is gene 1). The variation is mostly in the overall curvature of the epithelium. There is a default cell division rate in all cells. As in **a** cylinders represent epithelial cells, color represents z-axis coordinate values as in a topographic map. **d**. As in **c** but for the three final morphologies arising from variation in the diffusivity of the signal. The morphologies vary in how the curvature decreases with the distance to the signal’s source. In the morphology in the upper row the curvature is very strong near the cells secreting the signal. In the morphology in the lower row, the curvature is more evenly distributed (low row) (**e**) as in **a**. **f** As in **b** but for a different developmental mechanism. The gene that yellow cells in **d** express is gene 2. **g** Three examples of final morphologies arising from the initial morphology in **d** through the developmental mechanism in **e** (also according to EmbryoMaker)

Imagine an embryo consisting of a flat hexagonal epithelium in which all cells are identical except for cells in the anterior border (yellow cells in Fig. 2a). Imagine these latter cells express a gene that is not expressed elsewhere. Clearly, for this flat epithelium to change its morphology, cells have to change their position in space. This is because we define morphology as the distribution of cells in space and, thus, changes in morphology are changes in the position of cells in space. In other words, cells have to move, either actively or passively, for morphology to change. Cells move because they regulate cell behaviors that generate forces and movement or because they are bound to cells that move (also because of cells behaviors). The cell behaviors of animal cells are: cell division, cell growth, cell contraction, cell death, cell adhesion, ECM secretion, extracellular signal secretion and reception, with some small variations in this list depending on the author [43, 44].

For morphological transformations to occur, it is not enough that cells move. It is also required that cells in different places move in different directions. Otherwise we will simply get the same morphology (e.g. the flat epithelium) in a different position in space. Cells in different parts of an embryo can move in different directions even when cell behaviors are regulated in the same way by all cells in an embryo [12, 46]. In most embryos, however, cells in different places move in different directions because cell behaviors are differently regulated in different territories (i.e. territories can be understood as sets of cells with a specific distribution in space and some common gene expression) [12, 13, 47]. In turn, the formation of new territories requires a process of pattern formation in which signals are sent by some cells, transported over space and received by some cells [3]. Typically, the signals are gene products that are secreted into the extracellular space, passively diffuse and bind to specific membrane receptors. In the flat epithelium of Fig. 2, for example, cells in the anterior border secrete a diffusive signal. Due to the degradation of signals in the extracellular space and the physical process of diffusion, the concentration of this signal decreases with the distance to the signal’s source (i.e. the yellow cells in the anterior border, see Fig. 2a) [12]. This diffusion leads to the formation of at least two new spatial territories: the territory where the signal concentration is high enough to elicit a response by cells and the territory where it is not. Depending on the receptors and signal transduction networks expressed within cells, additional territories may form at different distances from the source of the signal [48].

In general, how cells respond to signals depends on complex networks of signal transduction and on the previous history of each cell [13]. These responses consist in the additional regulation of cell behaviors (e.g. secreting additional extracellular signals, cell division, etc.) and in changes in the expression of the genes involved in regulating these responses (e.g. signal receptors). As a result, cell collectives dynamically change their gene expression and new territories form over space and time. Each territory can differently regulate cell behaviors and, then, cells in different territories can move in different directions. In our flat epithelium, for example, cells respond to the diffusing signal by contracting their apical side with a force that is proportional to the concentration of the signal they receive. As a result, cells become more wedge-shaped close to the signal’s source and the epithelium forms an invagination whose curvature decreases with the distance to the signal’s source (see Fig. 2c).

This brief description of developmental dynamics highlights a simple fact: it is because cells interact and regulate their behaviors that morphology changes during development and morphological variation can occur. Gene networks are also required but their effect on morphology is mediated through their effect on these cell interactions and cell behaviors (cell signaling included). In the developmental dynamics depicted in Fig. 2, for example, the invagination of the epithelium requires apical cell contraction and the decrease in curvature over space requires a signal that diffuses in space and promotes apical cell contraction.

Here, as in [9], we use the term developmental mechanism for each gene network involved in a morphological transformation and the cell behaviors, cell mechanical properties and signals such a network regulates (see Fig. 2b,f for an example). From this terminology it follows that each morphological transformation requires a specific developmental mechanism (although a given morphological transformation may be possible from different developmental mechanisms [49]). Similarly, a morphology can vary in a specific direction if its underlying developmental mechanism can lead to morphologies in that direction In other words, there is no default morphological variation (i.e. as in the isotropic expectation); each direction of morphological variation is possible because of specific cell interactions and the regulation of cell behaviors. This implies that for a morphology to be able to vary in all direction, an extremely complex developmental mechanism with many gene products and involving the regulation of many different cell interactions, signals and cell behaviors would be required.

To clarify these points let’s consider the mutations that can occur in a developmental mechanism. Mutations can affect the intensity by which a gene product regulates other gene products, signals, cell behaviors and mechanical properties in a developmental mechanism. Mutations can also occur that change which gene products regulate which other gene products (including the recruitment of genes from outside the original developmental mechanism), signals, cell behaviors and cell mechanical properties. Here, as in [9], the first type of mutations will be called IS-mutations (from interaction strength) and the second type of mutation will be called topological or T-mutations because they change the topology of the network of a developmental mechanism.

The directions of morphological variation possible by IS-mutations are determined by the cell behaviors, cell interactions, gene product interactions and the arrangement of all those in a developmental mechanism. For example, a mathematical simulation of the developmental mechanism of Fig. 2b shows that there are two possible directions of morphological variation by IS-mutations: variation in the overall curvature of the epithelium or variation on how such curvature decreases with the distance to the signal source (see Fig. 2c). The first direction of variation arises from mutations affecting the amount of apical cell contraction (e.g. mutations in the second green arrow in the developmental mechanism of Fig. 2) while the second direction of variation arises either from changes in the rate of secretion of the signal or changes in the diffusivity of the signal. Decreases lead to the curvature to be higher close to the signal’s source, Fig. 2d higher row, while increases lead to a more evenly distributed curvature, Fig. 2d lower row. These are the only directions in which the morphology can vary due to IS-mutations in the developmental mechanism of Fig. 2b. Variation in other directions is not possible unless the developmental mechanism changes its structure, e.g. its topology, through T-mutations.

T-mutations can be expected to occur relatively rarely [9] and most natural populations would only exhibit a small number of alleles affecting the topology of a developmental mechanism in a population. Most T-mutations are unlikely to lead to new directions of morphological variation. Consider, for example, the flat morphology in Fig. 2. IS-mutations in its underlying developmental mechanisms lead to variation in the curvature of the epithelium but the curvature always decreases with the distance to the border of the epithelium (i.e. the signal’s source). A new direction of morphological variation would be, for example, that the curvature could increase at a distance from the margin. Figure 2f shows one of the simpler developmental mechanisms able to transform the flat epithelium into a morphology where the curvature can also increase at a distance from the signal’s source. As the figure shows, this developmental mechanism is quite different from the original one. Thus, for morphological variation to be possible in this new direction the underlying developmental mechanism requires a major restructuring, through many topological mutations. This implies that populations with the original morphology and developmental mechanism depicted in Fig. 2b would not exhibit variation in the new direction, or only very rarely, since this requires many T-mutations.

There is nothing special about the direction of morphological variation depicted in Fig. 2. Thus, in a natural population using a specific set of developmental mechanisms only a relatively small number of directions of variation would be possible. How small would be that number depends on the population, the mutation rate and the developmental mechanisms, but the previous arguments should make it clear that the probability of morphological variation being possible in all directions is absurdly small for any reasonably complex developmental mechanism.

There is an additional simple argument that makes the isotropic expectation untenable. Ultimately, all morphological transformations require cells to move and these movements arise from a limited small number of cell behaviors and the mechanical properties of cells and the ECM. Some authors argue that the morphological diversity found in animals can be decomposed into a relatively small set of basic shapes (e.g. tubes, cavities, condensations, etc. …). These are the shapes made possible by the cell behaviors and the mechanical properties of animal cells and the ECM [50]. In other words, the amazing morphological diversity of animals can be understood as the recombination, in space and time, of these basic shapes. Then, no morphology can vary in all directions, instead the directions of possible morphological variation are those compatible with these basic shapes. Certainly, cell behaviors could themselves vary and evolve to lead to new basic shapes, but this happens very rarely [51]. In fact, some authors [51], suggest that each time a new cell behavior has arisen in evolution, it has lead to a qualitative change in possible morphologies and to a major branching in animal evolution (e.g. porifera versus diploblasts, diploblasts versus triploblasts, etc.).

## The isotropic expectation and morphometrics

In many morphological studies, the morphological space is approximated by a tractable mathematical construct called morphospace [14, 52, 53]. A morphospace is a quantitative multidimensional space constructed from continuous or discrete morphological variables. Each distinct morphology corresponds to a point in a morphospace (see Fig. 1). Morphospaces have been widely used to discuss relevant topics in morphological evolution: developmental constraints [14, 52], functional morphology [54, 55], systematics [56], and evolutionary ecology [57, 58] among others. As noted by Gerber [33] many morphometric studies, especially those not directly related to developmental approaches, hold the view, implicitly, that the morphospace has a homogeneous and isotropic accessibility structure (sensu [34]). In other words, the view is that lineages, regardless of their location in the morphospace, can potentially evolve in every direction of the morphospace with equal facility. That is just another form the isotropic expectation takes. As we come to see, there is no actual reason why this should be the case. In other words, morphologies that are close in a morphospace may not be close developmentally (i.e. many changes in development may be required to lead from one to the other) [33, 34]. In fact, some authors have suggested that the variational properties of development can be used as the generative bases of morphospaces [33].

## The alternative: development-based expectations

A summary of the previous discussion is that the concepts of developmental constraints and bias have a number of shortcomings. First, they are based on an opposition to an expectation on possible morphological variation that is not always explicitly stated. Second, describing the role of development in evolution as a bias or constraint is only meaningful if the isotropic expectation is tenable, but it is not. Third, conceptualizing development as a bias or constraint does not fully acknowledge that development is the process that generates morphology and determines its patterns of variation.

Some of the early proponents [5, 14] of the developmental concept argued that developmental constraints can be understood in a negative way, as precluding some potentially adaptive morphology from existing, but also in a positive way, as facilitating some directions of morphological variation. Arthur even suggests using a different term, *developmental drive*, for the positive side of developmental constraints [8]. It should be noticed, however, that stressing the positive side of developmental constraints does not solve the shortcomings of the developmental constraints and bias concepts discussed in this article. As discussed in the previous sections, the developmental constraint and developmental bias concepts, either in its positive or negative meanings, imply there is an expectation on possible morphological variation (i.e. positive and negative imply a reference system with a zero) and that development is the cause for a departure from such expectation rather than the source of such expectation.

Gould conceived the positive side of developmental constraints in a slightly different way [19, 29]. For Gould every constraint entails a suite of nonadaptive consequences and these can be positive because they might later lead to some (perhaps novel) adaptation [19, 29]. Certainly, changes that are non-adaptive at a given time can become adaptive at some later time (e.g. the environment and then the selective pressures can change). However, underlying how Gould presents this idea, there is still the assumption that, by default, variation in all directions should be possible and, if that is not the case, development is one of the potential factors to blame.

If one acknowledges that development determines the possible directions of morphological variation, one should not use expectations on possible morphological variation that are not based on development itself. Instead, the role of development in evolution should be described by the morphological variation it can produce, not by the morphological variation it can not produce in respect to some expectation that is uninformed by our understanding of development and not especially plausible. In other words, expectations on possible morphological variation should be based on development itself, on what we understand about it or on reasonable hypotheses about it.

Most likely, the concept of developmental constraints has been crucial for a widespread realization that development is important for understanding evolution. Many current evolutionary biologists, especially in evo-devo, are acquainted with the idea that development is the process that determines which morphological variation is possible or that, at least, development has something to do with it. Perhaps it is then time to conceptualize the role of development in evolution directly, instead of indirectly as a departure from an old and no-longer tenable expectation. In other words, the “favored theory” against which Gould [19] defined constraints (see section 1) may have changed enough for development not being describable as the source of a departure from a favored theory. In fact, Gould himself [19] was already aware that, because of it the negative meaning, the concept of developmental constraint may be misleading and that it may be convenient to replace it for something else. Ultimately, however, he preferred to keep the concept and the discussion on its positive and negative meanings.

In the past I have proposed the concept of variational properties as a replacement for the concepts of developmental constraints and bias [9]. This concept can be applied to a developmental mechanism, to the development in an individual, population, species or set of species. The variational properties of a developmental mechanism are simply the morphological variation, i.e. the set of morphologies, that arise in it from IS-mutations or environmental changes. T-mutations can be considered to transform a developmental mechanism into another. This latter distinction is, however, not very important for the current discussion (see [9] for a detailed justification of this choice). What is important is that the concept of variational properties directly relates to the morphological variation development can produce. In other words, the variational properties of a development are an expectation on possible morphological variation that is based on development itself.

Although the variational properties concept may be useful, there is in fact, no need to replace the developmental bias and constraint concepts with any specific concept. If the aim is to argue that development matters to understand evolution because it determines which morphological variations is possible in each generation, one can simply explain that directly, either by this same sentence or by equivalent ones. Similarly, one can simply talk about “development” instead of “developmental constraints or biases” and talk about development leading to specific directions of morphological variation instead of development constraining or biasing morphological variation in respect to a no- longer-tenable expectation that is usually not stated explicitly. This is the reason for the title of this article. Similarly, if one wants to argue that an imagined adaptive morphology is not found in evolution because development cannot produce it, one can just state it in those same words (although being adaptive is not enough of a reason to expect that a morphology would arise in evolution).

These alternatives are simpler and more accurate than the developmental bias and constraints concepts. They keep the message of why development matters for evolution without having any of the shortcomings of the developmental constraints concept. First, they are not defined as an opposition to an expectation that is not tenable, in fact, they are not based on any expectation other than those coming from development itself. Second, they explicitly describe development as the process responsible for morphology and its patterns of variation rather than as a factor constraining or biasing it. In addition, as we describe in the last section of this article, these simpler concepts facilitate a different research program and research questions than the developmental constraint and bias concept.

## Development, variational properties and evolvability

The concept of variational properties describes the role of development in evolution based on the morphological variation it can produce. Some authors have proposed that the concept of evolvability could also provide a positive account of the role of development in evolution [18]. There are, however, important differences between the concept of variational properties and the concept of evolvability. The first one is that the concept of evolvability is understood and defined in different ways by different researchers [59]. A perhaps popular way to understand evolvability is as the capacity of a system to generate adaptive variation [60].

The concept of evolvability does not specify if it applies to adaptive variation at the genotypic or phenotypic level. Thus, for example, one can talk about the evolvability of genomes of different size [61], about the evolvability of the cytoskeleton [62, 63] or about the evolvability of abstract gene network properties (such as their interactions being weak) [62, 63], etc. This flexibility can be seen as an advantage since it facilitates the use of evolvability in different research areas (such as in evolutionary genetics [64] and in computer science [65]) or as an inconvenience, since different users of the concept may effectively mean different things [59]. In that sense, the concepts introduced in the previous section are much more concrete and precise since they apply only to the morphological variation arising from development.

Another difference is that evolvability focuses on adaptive variation (phenotypic or genotypic) while the concept of variational properties, and the other alternatives I proposed, focus on morphological variation irrespectively of its potential adaptive value. The problem is that what is adaptive, and what it is not, depends on the environment (i.e. natural selection) and the environment changes in time and space. In fact, how the environment changes is one of the main factors for understanding the direction of evolution [1]. Thus, by just looking at development, one cannot ascertain whether it is “evolvable”. In other words, the concept of evolvability collapses morphological variation and natural selection together. This is inconvenient since to understand the direction of morphological evolution one needs to first study which morphological variation is possible in each generation and, then, which of it is selected [46]. Moreover, one may also be interested in studying the morphological variation that is not adaptive and that, thus, is not considered by the concept of evolvability but is considered by the concept of variational properties. Because of those and other reasons I have, in the past, suggested that using the concept of variational properties is preferable to using the concept of evolvability [37].

## Example studies

Two case studies will be used to exemplify the above points. In an artificial selection experiment in butterflies [66], researchers selected for wings with larger anterior forewing’s eyespots that, at the same time, would have smaller posterior forewing’s eyespots (and vice versa). They found a clear response to selection and interpreted this result as an absence of developmental constraints and an evidence that wing morphology could be understood following the principles of population genetics and natural selection alone. However, the observed increases and decreases in the eyespots size, necessarily arose from changes in development since development is the process leading to the existence of these eyespots and their variation. In that sense development is also part of the explanation of why they got a response to selection in a specific direction.

Another popular set of studies that have been argued to be related to developmental constraints [27] are those of Raup’s on the morphology of mollusks shells. In Raup’s work [52, 67] a simple mathematical model of accretional growth and morphogenesis is used to predict the range of shell morphologies that should be possible in mollusks. He is, thus, not using the isotropic expectation on possible morphological variation but an expectation based on some understanding about shell development itself. He then asks why some of the morphologies that the model predicts are not observed in nature. Notice, he is not asking, as in the eyespot example, why some arbitrary morphologies are not observed. He is asking why some of the morphologies that are possible by development (i.e. from the model) are not actually observed in nature. Naturally then, he does not claim that these morphologies are not observed in nature because of a developmental constraint since he knows these morphologies should be possible by development. This is in fact, very similar to the approach that is proposed in this article: expectations on possible morphological variation should be based on what is understood about development.

## A different research program

From the perspective of variational properties and the other alternatives proposed in this article, the question is not whether development affects evolution (i.e. whether there are developmental constraints). The relevant question is how. More specifically the question is how the different ways in which development can work (e.g. different developmental mechanisms in different animals and body parts) lead to different morphological variation and, thus, affect morphological evolution differently. This leads to a research program that is different from the research program of developmental constraints and bias [3, 5, 14, 27]. In this section I outline some general evo-devo questions that arise from the perspective of variational properties. In principle, these questions could be addressed without the concepts proposed in this article, but these concepts naturally lead to ask these questions.

Given a set of morphologies (e.g. in a group of species) one can ask how does their development work and how the development of each morphology in the set differs from the development of the other. A large part of the research in evo-devo has always been devoted to this or similar questions. Most of this research has been at the macro-evolutionary level and has focused, mostly, on individual genes, gene expression or genetic interactions [7, 10, 68] rather than in developmental mechanisms per se. At the micro-evolutionary level of populations, there are fewer studies and they also tend to focus on individual genes, gene expression and some of their interactions [45, 69–72]. Only few studies focus on developmental mechanisms and morphological variation at the micro-evolutionary level. One exception is my study on teeth. By integrating current understanding on tooth development in a computational model of morphogenesis, we were able to reproduce the morphological variation of an adult 3D morphology in a natural population [73]. Using the model we also inferred which aspects of the underlying developmental mechanisms should be responsible for the observed morphological variation in the population (i.e. variation in which cell or gene interactions).

From the perspective of variational properties one can also address questions about natural selection itself. If the development of a morphology (e.g. the morphology of part of an individual) is well understood, it may be possible to predict the range of morphologies that would arise from it through IS-mutations (i.e. its variational properties). These can then be compared with the morphologies that are actually observed in a generation or set of generations in a natural population. If the observed morphologies are a small subset of the expected ones then one can infer that either natural selection has been stringent or that genetic drift has been important. Depending on the accuracy by which development is understood, evolution at different time scales could be studied. Unfortunately, our understanding of development is still not good enough for these kinds of approaches. In a similar but simpler approach the variational properties of the tooth development model were used to infer the extent to which natural selection can act on fine details of morphology [74]. This study suggests that, the genotype-phenotype map arising from the tooth model is quite complex and that given such complexity, tooth adaptation can only occur if the phenotype-fitness map is degenerate (i.e. many morphologies have the same fitness) [74].

Questions based on variational properties do not need to be based on reconstructing the past. Understanding the variational properties is understanding the morphological variation possible by development and this information can be used to make inferences on how morphology would evolve under different selective pressures and different developmental mechanisms. Thus, for example, we proposed a categorization of developmental mechanisms in animals and explained, by simulation [75, 76] and phylogenetic comparisons [77], how each type of developmental mechanism leads to different patterns of morphological evolution (e.g. different degrees of graduality and novelty, among others). Other authors propose other categorizations of developmental mechanisms that also have implications on the different ways in which morphology would evolve, in general [46–51, 78], or under different selective pressures [74–76, 79–84].

## Conclusions

When new theories arise, it is not uncommon that they are partially based on expectations and concepts from previous theories. The concept of developmental constraints can be seen this way: it was instrumental in highlighting the importance of development for evolution, but it is defined based on an expectation from a previous theory, the isotropic expectation of the modern synthesis. As theories further develop, these concepts, and the expectations on which they are based, may be superseded. It may then become useful to modify, replace or retire such concepts. Perhaps, then, it is time to replace the concepts of developmental constraints and bias with concepts that are not based on these superseded expectations but on the expectations coming from development itself. This should facilitate shifting the focus from the question of whether development matters for evolution to the question of how development matters for evolution.

## Data Availability

All data generated or analysed during this study are included in this published article and its supplementary information files.
